# Cardiac Masses: Pathological and Surgical Features - A Multicenter Study

**DOI:** 10.21470/1678-9741-2020-0225

**Published:** 2021

**Authors:** Arzu Taşdemir, Aydın Tuncay, Hatice Karaman, Ozlem Canoz, Ramazan Aşık, Rifat Özmen, Deniz Elcik

**Affiliations:** 1 Pathology Training Clinic, Kayseri City Hospital, Kayseri, Turkey.; 2 Department of Cardiovascular Surgery, Faculty of Medicine, Erciyes University, Kayseri, Turkey.; 3 Department of Cardiology, Faculty of Medicine, Erciyes University, Kayseri, Turkey.; 4 Department of Cardiovascular Surgery, Kayseri City Hospital, Kayseri, Turkey.

**Keywords:** Heart Ventricules, Heart Atria, Echinococcus, Neoplasms, Cardiovascular Diseases, Hospital Mortality

## Abstract

**Introduction:**

This study aimed to review the surgical excision results and pathological diagnostic features of rarely observed intracardiac masses in the light of the literature. Diagnosis and treatment approaches and complications were evaluated.

**Methods:**

Forty patients (26 females, mean age 52.1±18.1 years, and 14 males, mean age 48.1±20.5 years), who had undergone surgery for intracardiac mass between January 2008 and December 2018, were included in this study. The patients’ data were analyzed retrospectively from the medical records of both centers.

**Results:**

When the pathological diagnoses were examined, 85.8% of the masses (n=35) were observed to be benign (benign tumor + hydatid cyst) and 14.2% (n=5) were malignant tumors. The masses were most commonly located in the left atrium (75%, n=30), and this was followed by the right ventricle (12.5%, n=5), right atrium (7.5%, n=3), and left ventricle (5%, n=2). Of the patients, 7.5% (n=3) died during the early postoperative period, while the remaining 92.5% (n=37) were discharged with healing. In the histopathological diagnosis of the patients, in whom in-hospital major adverse cardiovascular events were observed, there was malignancy in two cases.

**Conclusion:**

Intracardiac masses, which have pathological features, are severe life-threatening problems. In-hospital mortality is frequent, especially in malignant tumors.

**Table:** 

Abbreviations, acronyms & symbols				
**ALT**	**= Alanine aminotransferase**		**IHC**	**= Immunohistochemical**
**AST**	**= Aspartate aminotransferase**		**LIMA-LAD**	**= Left internal mammary artery-left anterior descending artery**
**BUN**	**= Blood urea nitrogen**			
**CD**	**= Cluster of differentiation**		**TEE**	**= Transesophageal echocardiography**
**CK-MB**	**= Creatine kinase myocardial band**		**TTE**	**= Transthoracic echocardiography**
**COPD**	**= Chronic obstructive pulmonary disease**		**WBC**	**= White blood cells**
**CVO**	**= Cerebrovascular occlusion**			

## INTRODUCTION

Although benign and malignant cardiac tumors are rare, they are important causes of mortality and morbidity. Cardiac neoplasms are asymptomatic until they reach a large size, and present with nonspecific findings. Like myxomas, the origin of some of these tumors is uncertain. Cardiac masses have become a surgically treatable pathology with the introduction of the heart-lung pump. Malignant tumors of the heart could not get rid of the poor prognosis despite all advances.

The incidence of cardiac tumors is observed to be 0.02%, based on 22 large autopsy data from a United Kingdom centre^[1]^. In another autopsy series, from Germany, this rate is also shown in the range of 0.001-0.3%^[2]^. Primary tumors of the heart may be benign or malignant. Secondary tumors are metastatic and malignant tumors. Of all cardiac tumors, 75% are benign and 25% are malignant. Myxomas are the most common primary cardiac tumors^[3]^. In this study, we aimed to present the incidence, localization, histopathological diagnostic characteristics, and surgical approaches of intracardiac masses.

## METHODS

A total of 40 patients (26 females, 14 males), who had been operated in the Erciyes University Faculty of Medicine and Kayseri City Hospital Cardiovascular Surgery Departments, between January 2008 and December 2018 and whose postoperative tissues had been sent to the pathology clinic, were included in the study. The study was designed as a retrospective record review study. The approval of the local ethics committee was obtained.

All patients underwent parasternal long and short axis and apical four-chamber transthoracic echocardiography (TTE) (Vivid 7 GE) before the procedure. TTE was the common preoperative diagnostic method used in all patients for the location, size, and general characteristics of the mass. If necessary, transesophageal echocardiography (TEE) was performed under local anesthesia using a Vivid 7 GE device.

All patients underwent surgery under general anesthesia. A median sternotomy incision was performed in 39 patients. Only in one patient, diagnosed pathologically with myxoma, the mass was removed by preferring the right anterior thoracotomy incision. Following the classic median sternotomy, aortic cross-clamp and standard cardiopulmonary bypass and antegrade cardioplegia were performed together with aortic and bicaval venous cannulation. The incision performed for tumor resection was selected by considering the location of the tumor.

In hydatid cyst cases, saline-impregnated gauze was placed around the cyst so that the cyst content was not spilled outside. Some of the cyst content was aspirated with a syringe, and 3% serum sale was injected into it, and then the cyst content was carefully removed, and the cyst was cleaned. After this procedure, cystectomy + capitonnage was performed in all patients. While the defects formed after the removal of the mass were closed with primary sutures, the defect created after resection was closed by polytetrafluoroethylene in one patient with lipoma in the right atrium and by using a pericardial patch in two patients.

### Statistical Analysis

Statistically, the distribution of quantitative data was defined as x±standard deviation. The difference between the two groups was evaluated by Student’s t-test. Qualitative numbers were defined as percentage. The statistical significance level was set at 0.05.

## RESULTS

A total of 40 patients (26 females, mean age 52.1±18.1 years, and 14 males, mean age 48.1±20.5 years) were included in the study. The baseline characteristics of the patients are presented in Table 1. In terms of the location of the mass, the left atrium corresponds to 75% of all cases (30 cases), while the other lesions were determined in the right ventricle in five cases (12.5%), right atrium in three cases (7.5%), and left ventricle in two cases (5%). According to the recommendation of the European Society of Cardiology guidelines, coronary angiography was performed in nine patients who were over 40 years old and four patients who were postmenopausal women. In only one of these patients, the lesion to be treated was detected. Saphena-posterior descending artery and left internal mammary artery-left anterior descending artery (LIMA-LAD) anastomoses were performed simultaneously in this patient.

**Table 1:** Patients' baseline characteristics.

Variability	Number	%
Diabetes mellitus	4	8
Hypertension	2	4
Coronary artery disease	4	8
CVO	1	2
COPD	1	2
Hemoglobin (g/dl)	12.09 ± 2.31	
WBC (10^3µL)	10.9 ± 4.8	
Platelet count (10^3µL)	269.1±101.1	
Lymphocytes	1.8 ± 0.16	
Neutrophil	10.3 ± 2.5	
BUN	16.9 ± 13.3	
AST	31.2 ± 4.75	
ALT	26.6 ± 5.3	
CK-MB	42.6 ± 12.2	

ALT=alanine aminotransferase; AST=aspartate aminotransferase; BUN=blood urea nitrogen; CK-MB=creatine kinase myocardial band; COPD=chronic obstructive pulmonary disease; CVO=cerebrovascular occlusion; WBC=white blood cells

While left atriotomy was the preferred heart chamber in 19 cases, the transseptal incision was used to remove the mass in eight cases (Table 2).

**Table 2:** Site of surgical intervention and pathological diagnosis.

Intervention site	Pathological diagnosis	n	%
Left atriotomy	Myxoma	16	40
	Hydatid cyst	2	5.0
	Leiomyosarcoma	1	2.5
Transseptal intervention	Myxoma	4	10
	Diffuse cell lymphoma	1	2.5
	B-cell lymphoma	1	2.5
	Calcified amorphous tumor	1	2.5
	Cavernous hemangioma	1	2.5
Right atriotomy	Myxoma	3	7.5
	Hydatid cyst	1	2.5
	Squamous cell carcinoma	1	2.5
	Lemon	1	2.5
Right + left atriotomy	Myxoma	2	5.0
	Rhabdomyoma	1	2.5
Right ventriculotomy	Angiosarcoma	1	2.5
	Hydatid cyst	1	2.5
Left ventriculotomy	Hydatid cyst	1	2.5
Right atriotomy + ventriculotomy	Hydatid cyst	1	2.5
**Total**		**40**	**100**

**Fig. 1:**
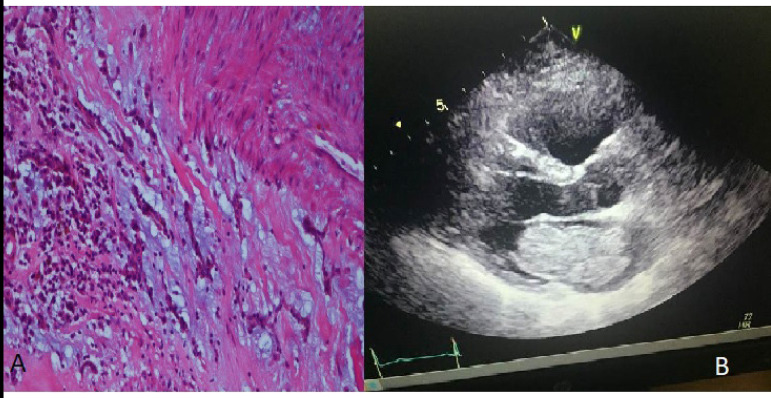
A) Histologically, the myxomas are composed of round, oval, polygonal, or stellate cells in abundant loose myxoid matrix containing abundant mucopolysaccharides. B) Echocardiography finding of left atrial myxoma.

And although 62% of the cases were myxomas (Figure 1 A and B), 12% were hydatid cysts. Other masses and their characteristics are shown in Table 3.

**Table 3:** Masses and rates.

Pathological diagnosis	n	%
*Myxoma*	25	62.5
Hydatid cyst	6	15.0
Angiosarcoma	1	2.5
Leiomyosarcoma	1	2.5
Rhabdomyoma	1	2.5
Calcified amorphous tumor	1	2.5
Metastasis of squamous cell carcinoma	1	2.5
Cavernous hemangioma	1	2.5
Lipoma	1	2.5
Lymphoma	2	5.0
Total	40	100

By considering the location of myxomas in the heart chamber, while the right and left atrium were opened separately together in two cases, the transseptal intervention was preferred in four cases. The created defects were repaired using a primary or pericardial patch. In one case, the mass in the left atrium was removed by preferring the right anterior thoracotomy incision.

Surgical interventions, saphena-posterior descending artery, and LIMA-LAD anastomoses were performed concomitantly in one patient in whom myxoma was excised in the right ventricle.

In another patient with left atrial myxoma, mitral valve replacement was performed at the same session upon observing the third mitral insufficiency after the intraoperative TEE.

Again, after the resection of the existing mass in the right atrium, a 4 x 4-cm mass in the right lung basal lobe posterior was removed by wedge resection during the same session. Both masses were diagnosed as a hydatid cyst.

**Fig. 2:**
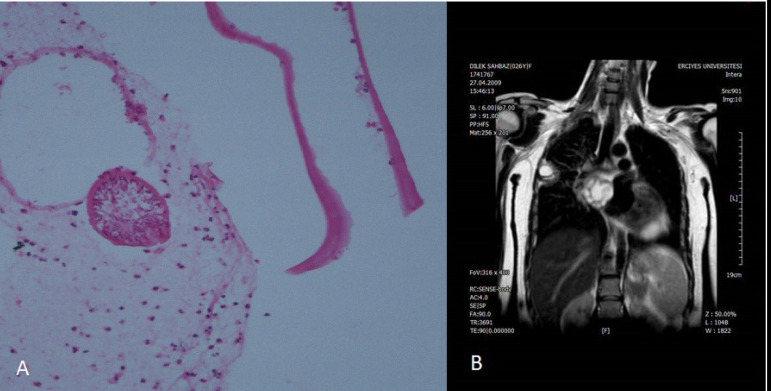
A and B) A cross section of a scolex surrounded by laminated membrane lining the hydatid cyst.

In another case, who presented with ischemia in the left lower extremity and whose left atrial mass was diagnosed as myxoma, thrombectomy was performed in the left lower extremity. The patient presented with a mass located in the left atrium diagnosed as a hydatid cyst, which is a nontumoral lesion in the pathology (Figure 2 A and B), after the mass was removed. In this patient, left hemiplegia was detected in the postoperative period.

**Fig. 3:**
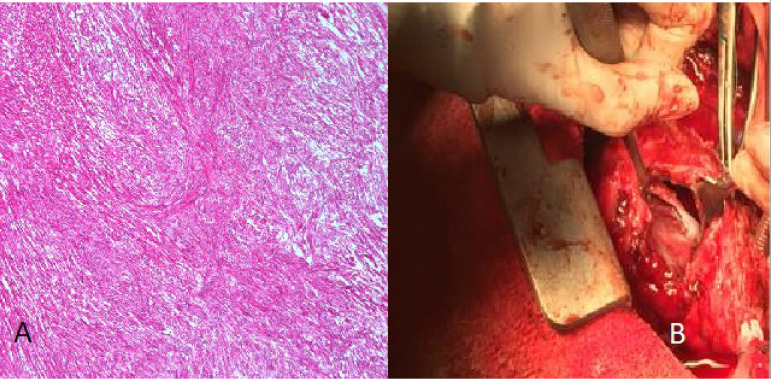
A) Compact bundles or fascicles oriented at right angles to each other. B) Right ventriculotomy procedure.

In another case reported as undifferentiated angiosarcoma (Figure 3 A and B), the right ventricle was completely cleaned, and the patient was discharged with healing. However, due to the recurrence of the symptoms 20 days later, the patient underwent TTE and was reoperated upon detecting a mass in the right ventricle. Intracranial hemorrhage was detected in diffusion magnetic resonance imaging, which was taken on the fourth postoperative day due to the deterioration of the condition, and the patient, therefore, died. This patient is our only relapse.

In another patient, who was six-month-old, perforation was detected in the region of the ileum close to the cecum during the postoperative period. The last part of the ileum, the cecum, the appendix, and the 5-cm segment of the colon were removed. The patient, in whom ileostomy and colostomy were performed, died in the early postoperative period.

Another patient, who was diagnosed with squamous cell carcinoma metastasis, was re-hospitalized on the eighth postoperative day due to the development of sternal infection, and the patient to whom sternal repair was applied was discharged with treatment.

The removed mass was diagnosed as glandular in three of our patients with myxoma and as a cellular variant in one of the patients. In all of these patients, it was noted that the location was the left atrium.

The number of early mortality cases in the postoperative period was three (7.5%), two of whom were from the malignant mass group.

## DISCUSSION

Cardiac tumors are rare and account for 0.2% of all tumors in humans. These are divided into two types, as primary and secondary/metastatic tumors. Secondary or metastatic tumors are 20-40 times more common than primary tumors. Primary intracardiac tumors are rare. Of the tumors, 75% are benign. Cardiac myxomas represent the most frequently observed primary cardiac tumors^[3-5]^; they make up about 50% of those and are observed more frequently in women of middle age.

Myxomas were reported to be in the left atrium in 75-80% of the cases, in the right atrium in 10-20% of the cases, and in the atrium and ventricle in 5-10% of the cases^[6]^. Myxomas are often observed as a single mass with a spherical shape and smooth surface originating from the left atrium endocardium, adjacent to the foramen ovale. However, macroscopically, they may have fingerlike protrusions, an irregular surface, and, sometimes, the appearance of a bunch of grapes. While 21 (84%) of the myxomas were located in the left atrium, two (8%) were in the right atrium and two (8%) in the right ventricle. Myxomas are a rare cause of acute peripheral arterial ischemia^[7]^. In our study, 60% of myxomas were detected on the left atrium side in accordance with the literature, and the majority were interfered with left atriotomy.

Myxomas may sometimes present with lower extremity embolism as the first symptom in the clinic. Among our cases, three patients were diagnosed with a glandular myxoma, and one was diagnosed with a cellular myxoma. It was remarkable that these four cases were located in the left atrium. Those, which are evaluated as a glandular myxomas, should be differentially diagnosed with metastatic adenocarcinomas. Myxomas are easily diagnosed pathologically with their typical histopathological appearance and classical locations. Although there is no statistically significant difference in myxomas in terms of glandular differentiation and prognosis, recurrence or metastasis has been reported in the literature^[8-10]^.

In the literature, there are studies showing that clonality may play a role in myxoma recurrences^[11]^. In a study, when the deoxyribonucleic acid was examined by the polymerase chain reaction method in tumor tissues after surgery, most of the cardiac myxomas were observed to be polyclonal. Their polyclonal origin characteristics may contribute to tumor recurrence^[11]^.

The treatment of myxomas is the surgical removal of the mass under cardiopulmonary bypass. The outcome of surgical treatment was excellent^[4]^. In all of our myxoma cases, during the excision of the mass, care was taken to remove it together with some part of the heart chamber where it was located. In the treatment of these patients, there was no need for additional treatment, and cure was provided with surgical treatment.

In the nontumoral hydatid cyst lesion, which is one of the causes of a mass in the heart, the most affected regions are, respectively, the liver, lung, brain, spleen, kidney, bone, muscle, pelvis, heart, spinal cord, and retina^[12]^. Cardiac echinococcosis is rare and occurs in 0.5-3% of all cases^[13,14]^. Clinical presentations are directly related to the location, size, and integrity of cysts and may vary from asymptomatic to sudden death^[15]^. In the microscopic examination, typical eosinophilic lamellar, with cuticular membranes, scolices are observed and can be easily pathologically diagnosed as macroscopic and microscopic. Even though the cardiovascular hydatid cyst is rarely observed, its early diagnosis and surgical management are essential^[16]^. In our cases, it was diagnosed echocardiographically as cystic lesions and was immediately operated. As a result, successful resections were made, and mortality was not observed. In our study, 12% of total cases formed hydatid cysts and most of these cases were detected in the left system.

Cardiac lipomas are observed rarely and mostly consist of benign tumors, frequently remaining asymptomatic during a patient’s lifetime. These tumors are usually located in the epicardium and cardiac cavities. Interatrial lipomatous hypertrophy found in the pathological differential diagnosis is a rarely observed non-capsular hamartomatous lesion and is also called as lipomatous hypertrophy of the atrial septum. Even though the resection of cardiac lipomas is mostly easily performed, a small number of patients with extensive spread, unfortunately, undergo a difficult surgery, having poor outcomes^[17]^. The case of lipoma in our series was successfully resected, and no complication developed.

Hemangioma of the heart, which presents as a primary cardiac tumor, is sporadic and constitutes about 2% of all primary cardiac tumors^[18]^. A cardiac hemangioma may emerge at every age, in any localization, such as the pericardium, endocardium, or the myocardium in the heart. The histological subtypes of cardiac hemangioma are as follows: cavernous hemangioma, capillary hemangioma, and arteriovenous hemangioma. The cavernous hemangioma consists of multiple dilated vessels with thin-thick walls. Surgery is the best way to treat cardiac hemangioma^[18]^. The prognosis after surgery is good.

Rhabdomyomas, which constitute 60-86% of fetal cardiac tumors, are associated with tuberous sclerosis at a rate of 52-79%. The tumor is considered to be a hamartoma instead of a true neoplasm^[19]^. They are well-circumscribed, solid masses, which are either grown into the cavity or embedded into the myocardium and they are often numerous. From a histological aspect, every mass is composed of large cells with clear cytoplasm-spider cells, which take their name due to their radial cytoplasmic strands. Rhabdomyomas are spontaneously regressing tumors at a rate of 50-100%. Therefore, clinical follow-up is recommended for asymptomatic patients^[20]^. One of our cases was a case of rhabdomyoma located in the left atrium.

Malignant heart tumors originate from the myocardium, endocardium, epicardium, and pericardium. The pericardium is frequently the localization of metastases. Of the malignant tumors, about 75% are sarcomas^[21]^. The most common sarcomas are angiosarcoma and undifferentiated pleomorphic sarcoma. Angiosarcoma, the most common type of sarcoma, is located most frequently in the right atrium in all age groups^[22]^. The pericardium is frequently involved. Cardiac tamponade and pericardial effusion are common complications. They are often not well-circumscribed, hemorrhagic, and appear as necrotic masses. Histopathologically, the vascular channels formed by atypical, pleomorphic endothelial cells attract attention. Occasionally, the areas of hemorrhage and necrosis and mitotic figures accompany them. Histopathology analyses must contain immunohistochemical (IHC) stain, and a large panel of antibodies should be carried out for the purpose of avoiding misdiagnosis. Angiosarcomas are positive for vascular markers, including cluster of differentiation (CD) 31 and CD34. Most tumors are poorly undifferentiated. Cardiac angiosarcomas frequently have a poor prognosis, even with treatment^[23]^. They usually affect men at the age of 40 to 50 years, and they have a worse prognosis in patients at a younger age. In our study, a patient diagnosed with undifferentiated angiosarcoma was examined and the tumor proliferation index was found to be high in Ki67 IHC staining. Twenty days after the complete removal of the mass by operation, the patient applied to cardiac surgery, and the mass was cleaned again through reoperation. However, the patient died due to intracranial hemorrhage despite all efforts.

Cardiac leiomyosarcoma constitutes less than 1% of cases. The median age at diagnosis was 48 years with a female predominance. Cardiac leiomyosarcomas are most frequently located in the left atrium^[24]^.

In the pathological diagnosis, the tumor was observed to be of blunt, fusiform appearance, and as bundles constituted by cells, of which cytoplasm boundaries were not clearly selected. Necrosis, hemorrhage, and myxoid degeneration areas may be observed between the tumor cells. Meanwhile, pleomorphic cells, osteoclast-type giant cells, and increased mitotic activity may accompany them. In IHC examinations performed for differential diagnosis, while vimentin, smooth muscle actin, myoglobin, and positive reaction in tumor cells are expected, a negative result is obtained with factor VIII-related antigen, epithelial membrane antigen, and CD34.

Leiomyosarcomas are tumors with a poor prognosis and have a frequent recurrence and distant metastatic potential. The primary treatment is the complete excision of the mass with a clear surgical margin, and with the multidisciplinary approach, chemotherapy and/or radiotherapy can be used palliatively in some cases. In our series, the diagnosis is leiomyosarcoma, located in the left ventricle in a 39-year-old male patient. The patient was evaluated at the oncology council and chemotherapy was planned. After the extensive surgical excision of the mass, the patient died before starting chemotherapy.

Primary cardiac lymphoma represents a malignancy, which is observed very rarely^[25,26]^. It is usually of a non-Hodgkin type and includes only the heart and pericardium. Although more than 20% of the patients with nodal, extra-nodal, disseminated lymphoma may have cardiac involvement, it may also be observed as lymphoproliferative diseases in immunosuppressive patients. Approximately 80% of primary cardiac lymphomas in immunocompetent hosts are diffuse B-cell lymphomas, and those are found in subjects with immunodeficiency states. The right atrium and right ventricle are their frequent localizations. Clinical presentation is heterogeneous and is usually associated with the site of involvement in the heart^[26]^. Histopathological diagnosis is the main diagnostic method, and typification requires a large IHC staining panel such as CD20, CD5, CD3, CD10, B-cell lymphoma 2 protein, and Ki67. Chemotherapy has been utilized alone or in combination with radiotherapy. Palliative cardiac surgery has been carried out, primarily for the purpose of tumor debulking. The combination of chemotherapy and radiation therapy is accepted to be the treatment of choice. The survival is usually less than a month without treatment^[25,26]^. In the series we presented, there are two malignant B-cell lymphoma cases. Two patients died without chemotherapy.

Metastatic tumors in the heart are more commonly observed than primary tumors. Metastatic tumors to the heart are observed at least 100 times more frequently in comparison with primary cardiac tumors when identified at autopsy, occurring in 1-3% of all autopsied patients^[4,5]^. In the presence of metastatic tumors in the heart, metastatic foci are also usually found in other localizations. In very rare cases, the heart is the first and only metastatic focus. Pericardial involvement is frequently observed. Lung, esophagus, melanoma, breast, renal cell carcinoma, choriocarcinoma, and childhood rhabdomyosarcomas are the most common metastatic tumors in the heart. Head and neck cancers rarely cause distant metastasis, involving the heart. In our series, there is a case of squamous cell carcinoma, of which the primary region is the head and neck region.

### Limitations

The most important limitations of this study are the small number of patients and the small number of centers.

## CONCLUSION

Intracardiac masses, which have pathological features, are severe life-threatening problems.

It is inevitable that masses in the heart, which is a vital organ, will be treated as privileged without making a benign-malignant distinction. The signs and symptoms of cardiac masses depend more on their localization than a histological diagnosis. In principle, patients who are eligible for surgery are treated urgently with the risk of sudden death and embolism in mind. In particular, malignant tumors involve severe mortality.

**Table:** 

Authors' roles & responsibilities	
AT	Substantial contributions to the conception or design of the work; or the acquisition, analysis, or interpretation of data for the work; drafting the work or revising it critically for important intellectual content; final approval of the version to be published
AT	Substantial contributions to the conception or design of the work; or the acquisition, analysis, or interpretation of data for the work; drafting the work or revising it critically for important intellectual content; final approval of the version to be published
HK	Substantial contributions to the conception or design of the work; or the acquisition, analysis, or interpretation of data for the work; drafting the work or revising it critically for important intellectual content; final approval of the version to be published
OC	Substantial contributions to the conception or design of the work; or the acquisition, analysis, or interpretation of data for the work; drafting the work or revising it critically for important intellectual content; final approval of the version to be published
RA	Substantial contributions to the conception or design of the work; or the acquisition, analysis, or interpretation of data for the work; drafting the work or revising it critically for important intellectual content; final approval of the version to be published
RÖ	Substantial contributions to the conception or design of the work; or the acquisition, analysis, or interpretation of data for the work; drafting the work or revising it critically for important intellectual content; final approval of the version to be published
DE	Substantial contributions to the conception or design of the work; or the acquisition, analysis, or interpretation of data for the work; drafting the work or revising it critically for important intellectual content; final approval of the version to be published
